# How does independent director affect tunneling?—Evidence from social networks

**DOI:** 10.3389/fpsyg.2022.1011761

**Published:** 2022-11-10

**Authors:** Hanxiu Cheng, Jie Wang, Mu Xing

**Affiliations:** School of Economics and Management, Nanjing University of Science and Technology, Nanjing, China

**Keywords:** social networks, tunneling, corporate governance, independent director, controlling shareholders

## Abstract

Given the influence of controlling shareholders on the company, it is important to analyze how independent directors can protect minority shareholders’ interests using the information and resources obtained from social networks. This paper studies the impact of director networks on controlling shareholders’ tunneling behavior in China over the period 2002–2020. Using social network analysis, this paper finds that controlling shareholders’ appropriation to minority shareholders is mitigated in companies with well-connected independent directors. These results remain consistent after a series of robustness and endogeneity tests. This study also reveals that internal controls play a mediating role between director networks and tunneling behavior. In addition, the study indicates that the restraining effect of director networks on controlling shareholders’ tunneling behavior is more pronounced in companies with weaker audit monitoring and poorer transparency. In conclusion, the results reveal that well-connected independent directors play an important role in protecting minority shareholders’ interests.

## Introduction

This paper examines how director networks impact controlling shareholders’ tunneling behavior. Director networks provide an informal channel for independent directors to communicate with each other among different companies. By serving on multiple companies’ boards, independent directors can not only access the latest trends related to corporate governance and development but also pass on the information to the companies located in director networks. Previous studies have shown that companies with well-connected independent directors typically exhibit higher quality financial reporting (Omer et al., 2020), higher stock returns (Larcker et al., 2013), and lower stock price crash risk (Fang et al., 2021). However, relatively few studies have considered how the network connectivity of independent directors affects their corporate governance capabilities. This paper examines the relationship between independent directors’ network connectedness and controlling shareholders’ tunneling behavior.

It is important to clarify how independent directors with connections to other firms’ boards can impact controlling shareholders’ tunneling behavior. Tunneling is an opportunistic behavior in which controlling shareholders use their controlling power to transfer the company’s assets and profits to their own (Johnson et al., 2000) which seriously undermines the minority shareholder’s interests as well as exacerbates the Type II agency conflict. Especially in countries with weaker investor protection, tunneling behavior is more prominent (Friedman et al., 2003). Independent directors are generally considered to bear greater responsibility for protecting the interests of minority shareholders. In 2004, the China Securities Regulatory Commission issued “Certain Provisions on Enhancing the Protection of Public Shareholders’ Rights and Interests,” which emphasized that independent directors should be independent in performing their duties, free from any influence by the actual controlling shareholders. Moreover, for material related party transactions, the approval of all independent directors is required. While there is considerable doubt about independent directors’ effectiveness in performing their duties (Morck, 2004; Ye, 2014; Wang, 2015), they do have an indispensable role in corporate governance. Tunneling behavior severely restricts the companies’ development and harms minority shareholders’ interests. Well-connected independent directors may effectively monitor controlling shareholders’ tunneling behavior. Typically, tunneling behavior is extremely insidious. Independent directors use the information gained from director networks and governance experience accumulated through serving in several companies to identify the self-interested behavior of controlling shareholders. Moreover, well-connected independent directors usually have a higher reputational cost (Ferris et al., 2003; Renneboog and Zhao, 2011). Independent directors who are at the center of the network show higher independence and are more able to say “No” to controlling shareholders’ tunneling behavior. Accordingly, the advantage of obtaining well-informed is essential for independent directors to perform their corporate governance duties.

While rich networks of directors contribute to the information exchange, it may also adversely affect corporate governance. On the one hand, according to the director busyness theory, independent directors who are in several companies may be less effective in monitoring the company due to busyness (Fich and Shivdasani, 2006; Falato et al., 2014). On the other hand, independent directors’ extensive exchange of information between different companies also carries a risk of disclosing firms’ sensitive information (Akbas et al., 2016; Ahern, 2017; Berkman et al., 2020). Therefore, it is unclear whether well-connected independent directors have a positive or negative impact on controlling shareholders’ tunneling behavior.

Chinese capital market is an ideal research context for studying how well-connected independent directors affect controlling shareholders’ tunneling behavior. First, China is an emerging capital market with a relatively weak legal system that protects investors, which facilitates controlling shareholders to expropriate minority shareholders’ interests. Second, shareholdings in Chinese listed companies are very concentrated, and controlling shareholders hold a great deal of power in the company. However, controlling shareholdings are restricted greatly in trading, which increases the possibility of controlling shareholders benefiting from tunneling behavior. Scholars such as Cheung et al. (2009), Jian and Wong (2010), and Jiang et al. (2010) have provided evidence that controlling shareholders widely engage in tunneling to profit among Chinese listed companies. Third, China is a humanitarian society. Networking and maintaining relationships profoundly affect individual behavioral decisions. Independent directors develop broad relationships that have the potential both to promote greater independence in corporate governance and to avoid disapproving behavior by maintaining relationships. Therefore, it is an interesting and worthwhile topic to investigate how the different degrees of relationships established by independent directors affect their decision-making in corporate governance when facing the common legal system environment.

Drawing on the sociological network centrality analysis, this paper constructs director networks for each year from 2002 to 2020 using a sample of Chinese A-share listed companies and calculates independent directors’ network centrality. The paper constructs four centrality metrics (degree centrality, closeness centrality, betweenness centrality, and eigenvector centrality) and measures the composite score *via* principal component analysis. The composite score is used in this paper to measure the firm-level director networks’ connectedness. Firstly, this paper examines the relationship between director networks and controlling shareholders’ tunneling behavior. It finds that the appropriation of minority shareholders by controlling shareholders decreases as director network connectedness increases, in line with predictions. After a series of robustness and endogenous tests, the conclusions remain the same. Second, the paper also finds the mediating role of internal controls between director networks and controlling shareholders’ tunneling behavior. To test whether the negative relationship between director networks and tunneling behavior is influenced by the corporate governance environment, the following tests are conducted: (1) examine the variation of main regressions under the different extent of audit monitoring; (2) examine the effect of firm transparency on main regressions. The results show that the negative relationship between director networks and controlling shareholders’ tunneling behavior is more pronounced in firms with low audit monitoring and poor transparency. In conclusion, this study provides evidence that, with the information and resources obtained through the network, independent directors can effectively monitor controlling shareholders’ opportunistic behavior and protect minority shareholders’ interests.

This paper makes several contributions to existing literature. First, this paper contributes to the literature on the determinants of controlling shareholders’ tunneling behavior. Previous literature has examined the impact of independent directors on tunneling behavior. For example, Liu et al. (2016) found that independent directors’ attendance at board meetings helped mitigate tunneling behavior. Gao and Kling (2008) and Gong et al. (2021) provided evidence that the proportion of independent directors was associated with less tunneling behavior. This study demonstrates how well-connected independent directors play an important role in mitigating controlling shareholders’ tunneling behavior. The study differs from Chen et al. (2014), as this paper provides evidence of the mechanisms by which director networks influence tunneling rather than just demonstrating the relationship between director networks and tunneling behavior. That is, this paper proves that well-connected independent directors improve internal control quality, which in turn inhibits controlling shareholder tunneling behavior. Second, this study contributes to the corporate governance literature using social network theory. Previous research has found that external connections of independent directors can bring good information to a company and improve the quality of financial reporting (Omer et al., 2020). It could also potentially disclose sensitive company information and result in more insider trading (Akbas et al., 2016). This paper provides evidence that well-connected independent directors play a positive role in monitoring controlling shareholders’ opportunistic behavior. Moreover, the governance role of director networks is more pronounced in the context of weak external audit monitoring and low corporate transparency.

This paper is arranged as follows. In Section 2, the paper reviews prior literature and proposes the hypothesis. Section 3 describes the data sample and research design. Section 4 performs the main hypothesis on the relationship between director networks and controlling shareholders’ tunneling behaviors. Section 5 provides a mediating effects analysis. Cross-sectional analysis is performed in Section 6. The conclusions are summarized in Section 7.

## Literature review and hypotheses development

### Controlling shareholders’ tunneling behavior

Controlling shareholders could appropriate minority shareholders’ interests in various ways, such as transferring company assets through related-party transactions (Aharony et al., 2010; Jian and Wong, 2010), providing loan guarantees for controlling shareholders (Jiang et al., 2010), malicious dividends (Chen et al., 2009; Atanassov and Mandell, 2018), etc. Johnson et al. (2000) called them “Tunneling,” which referred to the self-interested behavior in which controlling shareholders transfer assets and profits from companies. Especially in countries with underdeveloped capital markets (Friedman et al., 2003), the weak legal protection system for investors gives controlling shareholders more opportunities to manipulate minority shareholders’ interests (Cheung et al., 2006). The tunneling behavior has serious negative effects, both on the interests of minority shareholders and the performance of the company. Jiang et al. (2010) observed that controlling shareholders extensively used company loans to transfer assets from listed companies, which seriously undermined the company’s operating performance. Chen et al. (2011) revealed that controlling shareholders’ tunneling behavior had a long-term negative impact on the companies’ stock return. Chan et al. (2016) provided evidence that although previous studies have found that tax avoidance activities increased firm value when tax avoidance activities were associated with tunneling, the firm value was discounted. Although controlling shareholders’ opportunistic behavior seriously undermines companies’ and minority shareholders’ interests, Cheung et al. (2006) and Aharony et al. (2010) found it extremely secretive and difficult to predict in advance. Atanassov and Mandell (2018) stated that good corporate governance would help to alleviate controlling shareholders’ appropriation. Effective internal controls (Ge et al., 2021), strengthened internal governance by independent directors (Chen et al., 2014), and outside audit monitoring (Jiang et al., 2010) could also mitigate controlling shareholders’ tunneling behavior.

### Director networks

Previous studies supported the view that independent directors with extensive networks are better at monitoring companies. Carpenter and Westphal (2001) observed that independent directors’ social networks contribute to the firms’ strategic decisions. Using the high-quality resources provided by director networks, companies can more easily pursue new growth opportunities (Larcker et al., 2013; Singh and Delios, 2017). Well-connected independent directors accessed additional information and resources by serving on multiple company boards, which not only helped to reduce the likelihood of misstatements in financial reporting (Omer et al., 2020) but also helped to improve management’s earnings forecasting accuracy (Schabus, 2022). In addition, Li et al. (2019), Field et al. (2013) and Feng et al. (2019) have shown that independent directors located at the central location of the director networks played an important role in enhancing the efficiency of financing and improving IPO valuation.

While serving on multiple boards increases the likelihood that independent directors provide information and resources to the company, the opposing views argue that it can also divert their efforts and reduce the efficiency of monitoring (Core et al., 1999; Falato et al., 2014; Liu et al., 2022). For example, Fich and Shivdasani (2006) provided evidence that independent directors were busy due to multiple directorships and that the companies they serve exhibit lower profitability. Both Core et al. (1999) and Fich and White (2003) found evidence that there was a positive relationship between independent directors occupying multiple board positions and CEO compensation. On the other hand, the concern about director networks is that well-connected independent directors may inadvertently leak sensitive company information. Akbas et al. (2016) revealed that for companies with well-connected directors, investors were more informed. Ahern (2017) further proved that social networks provided useful information for insider traders. In addition, Berkman et al. (2020) observed that directors also benefited from the information advantage in the director networks and had more shareholdings and transactions in companies with interlocking relationships. In conclusion, there is no consistent agreement on the effectiveness of director networks. While the resources and information that directors access through networks can enable them to be strict monitors, there are also potential negative consequences for the company due to their busy directorships.

Taken together, the existing studies have not reached a consistent conclusion on whether external connections of independent directors can improve corporate governance quality. Independent directors’ extensive external connections reflect both resource accessibility and work busyness. Therefore, it is difficult to distinguish the impact of director networks on independent directors’ corporate governance capabilities. Controlling shareholders enjoy greater power in the company and are likely to influence independent directors’ appointments. For this reason, independent directors may succumb to controlling shareholders’ pressure and ignore tunneling behavior. However, well-connected independent directors, driven by reputation, are likely to maintain a high level of independence and actively perform monitoring duties. This paper attempts to advance the evidence on director networks and corporate governance capacity by examining how director networks affect controlling shareholders’ tunneling behavior.

### Hypothesis

In the Chinese capital market, a high concentration of shareholdings in listed companies leads to increasingly prominent conflicts between controlling shareholders and minority shareholders. Controlling shareholders gain private interests by transferring listed companies’ assets through related party transactions, earnings management, and other inappropriate means, described as “tunneling” (Johnson et al., 2000). It seriously damages minority shareholders’ interests (La Porta et al., 2000; Cheung et al., 2009; Jian and Wong, 2010; Jiang et al., 2010). As the primary monitoring mechanism, regulatory authorities hold high expectations for an independent director to perform effective monitoring functions to protect the minority shareholders’ interests. In 2001, the China Securities Regulatory Commission (CSRC) issued the “Guiding Opinions on the Establishment of Independent Director System in Listed Companies” which stipulated that independent directors should express an independent opinion on “Whether shareholders, actual controllers, and their affiliates of the listed company have existing or newly incurred loans or other financial transactions to the listed company with a total amount higher than 3 million RMB or higher than 5% of the latest audited net asset values of the listed company, and whether the company has taken effective measures to collect the debts.” In 2004, the CSRC issued “Regulations on Strengthening the Protection of Public Shareholders’ Interests,” which emphasized again that significantly related party transactions should be approved by more than half of the independent directors. Based on resource dependency theory, independent directors are an important channel to connect the company with the external environment, and their human and relational capital acquired *via* network connections is an important mechanism for the company to deal with external uncertainty (Hillman et al., 2000). The more central the independent director is in director networks, the more information they obtain about the industry, strategy, risk, etc. As a result, well-connected independent directors contribute to the quality of board decisions, which in turn restrain controlling shareholders’ tunneling behavior. As discussed above, although the evidence on the effectiveness of director networks is mixed, this paper conjectures that well-connected directors are effective in restraining controlling shareholders’ tunneling behavior. The discussion above is formalized into hypotheses, as follows:

*Hypothesis*: Director networks are negatively related to controlling shareholders’ tunneling behavior.

## Research design

### Data and sample

The sample consists of A-share listed companies on the Chinese market during the period 2002–2020. The sample period begins in 2002 as the independent directorship in China is mandatory from 2002, and ends in 2020, the latest year for which full data are available to measure the one-year lagged variable. This paper mainly obtains data from the China Stock Market and Accounting Research (CSMAR) database. The internal control data are from the DIB Internal Control and Risk Management Database. In line with prior studies, companies in the financial industry and observations with missing data are excluded. Table 1 shows the selection process of the sample. Ultimately, this paper obtains 31,826 firm-year sample observations.

**Table 1:** Sample description.

**Panel A: Sample selection for constructing director networks (2002–2020)**	
Director-year observations	
Observations of personal characteristics of directors, supervisors and executives	897,333
Less observations of non-board members	(476,008)
Less directors appearing twice in the same firm and year	(25,113)
Sample used to construct the director networks	396,212
Less observations of non-independent directors	(253,441)
Sample used to calculate company-level director networks	142,771
**Panel B: Sample for regression analysis**	
Firm-year observations	
Firm-year observations for the sample after merging with other data	40,676
Less observations in the financial industry and missing values	(8,850)
Firm-year observations used for regression analysis	31,826

### Empirical model and control variables

To test hypothesis, this paper estimates the following multivariate regression model to test the effect of director networks on different forms of tunneling:


(1) $$\begin{array}{c} Tunnel_{i,t}=\beta_{0}+\beta_{1}Networks_{i,t}+Controls_{i,t}+\\ \sum Year+\sum Industry+\varepsilon_{i,t} \end{array}$$

where the dependent variable, *Tunnel*, is measured by other receivables divided by total assets (Jiang et al., 2010; Liu et al., 2016). The independent variable, *Networks*, is the comprehensive measure of director networks. The specific calculation steps are described in subsection 3.3. The median of independent director centrality indicators is used to measure firm-level centrality indicators and conduct principal component analysis to construct firm-level network connectedness (*Networks_media*). The mean (*Networks_ mean*) and maximum values (*Networks_ max*) are used for robustness analysis.

This study controls for a set of variables in all specifications: the natural logarithm of the total assets at the end of the year (*Size*); the net income divided by total assets at the end of the year (*ROA*); growth in sales from the last fiscal year to the current fiscal year (*Growth*); an indicator variable equal to one if company report income less than zero (*Loss*); total debt divided by total assets at the end of the year (*Lev*); current assets divided by current liabilities (*CurrentRatio*); an indicator variable that takes the value of 1 if the board chairman and CEO are the same people (*Dual*); the percentage of independent directors on the board (*Ratio*); the book value of equity divided by the market value of equity (*BTM*); an indicator variable equal to one if a company is state owned (*SOE*); shareholding of institutional investors (*InvestorShare*); an indicator variable equal to one if the company is audited by a Big 4 audit firm (*Big4*). All variables are winsorized at 1 and 99%. The industry-fixed effects and year-fixed effects are also controlled. Detailed definitions of the variables used in this paper are provided in Appendix A.

### Measurement of director networks

Following previous research on director networks (Larcker et al., 2013; Omer et al., 2014, 2020), this paper uses four centrality measures, degree centrality (*Degree*), closeness centrality (*Closeness*), betweenness centrality (*Betweenness*), and eigenvector centrality (*Eigenvector*), to measure different aspects of director networks and use principal component analysis to form a composite measure.

***Degree centrality*** indicates that one director is directly connected to other board members in the director networks. If two independent directors work for the same board in year *t*, the companies are linked together through this director, forming an inter-company network. The degree of centrality measures how important the independent directors are in the network. The formula for degree centrality is as follows,


$$Degree=\frac{\sum_{i\neq j}\delta \left(i,j\right)}{n-1}$$

where *i* indicates an independent director, *j* is a board member other than *i* in the board networks. δ(i,j) equals to 1 if board *i* and board *j* work together on at least one corporate board, and 0 otherwise. *n* indicates the number of board members in the entire board network. The number of people in board networks varies every year, and n−1 is introduced to eliminate the effect of network size (Freeman 1978).

***Closeness centrality*** represents the close relationship among individuals. If a director can quickly connect with others in the network, which means that the director has faster access to information or resources (Larcker et al., 2013), then the director has a high degree of closeness centrality. The formula for closeness centrality is as follows,


$$Closeness=\frac{n-1}{\sum_{i\neq j}\mu \left(i,j\right)}$$

where μ(i,j) is the shortest distance from director *i* to director *j*.

***Betweenness centrality*** measures the ‘bridging’ role that a director plays in the director networks. If a director is located in the paths connecting various directors, it means that the director has informational or relational importance in the director networks. The specific calculation of betweenness centrality is as follows,


$$Betweenness=\frac{2}{\left(n-1\right)\left(n-2\right)}\underset{i\neq j\neq k}{\sum }\frac{g_{i}\left(j,k\right)}{g\left(j,k\right)}$$

where $g_{i}\left(j,k\right)$ is the number of shortest paths that director *j* connects to director *k* through director *i*. g(j,k) refers to the number of shortest paths connecting director *j* to director *k*.

***Eigenvector centrality*** describes the quality of network relationships. The degree of centrality measures the direct connection of the director in the network. Whereas, eigenvector centrality measures whether the neighbors of that director are well-connected. Following Bonacich (1987), eigenvector centrality is calculated as follows,


$$Eigenvector_{i}=\frac{1}{\lambda }\underset{j}{\sum }g_{ij}E_{j}$$

where $g_{ij}$ is the adjacency matrix, and $g_{ij}$ equals 1 if director *i* and director *j* work on at least one board, and 0 otherwise. $E_{j}$ is the eigenvalue of the centrality of director *j*. λ is the maximum eigenvalue of the adjacency matrix. The matrix form is as follows,


λE=GE

Higher eigenvector centrality of independent directors means they are more prestigious in the network and more advantageous in accessing information and resources in the network (Larcker et al., 2013).

For each company, the study calculates the median of all director centrality indicators as the firm-level centrality. Meanwhile, robustness analysis is performed using the mean and maximum values. Each centrality indicator measures a different aspect of the director networks, and it is unclear which particular indicator better captures their economic value (Larcker et al., 2013; Omer et al., 2020). Moreover, as shown in Panel A of Table 2, the four centrality indicators are highly correlated. Therefore, this research cannot simply use one indicator to measure director networks’ connectedness.

**Table 2:** Correlation analysis between centrality indicators.

**Panel A: Correlation analysis of the four centrality indicators**				
	*Degree*	*Closeness*	*Betweenness*	*Eigenvector*
*Degree*	1.000			
*Closeness*	0.257^***^	1.000		
*Betweenness*	0.710^***^	0.367^***^	1.000	
*Eigenvector*	0.092^***^	0.064^***^	0.080^***^	1.000
**Panel B: Principal component analysis**				
	Comp1	Comp2	Comp3	Comp4
*Degree*	0.6167	−0.0857	−0.4003	0.6723
*Closeness*	0.4289	−0.0433	0.8925	0.1325
*Betweenness*	0.6442	−0.1104	−0.2068	−0.7281
*Eigenvector*	0.1440	0.9892	−0.0187	−0.0172
*Eigenvalue*	1.942	0.980	0.798	0.279
*Proportion* (%)	0.486	0.245	0.200	0.069
*Cumulative* (%)	0.486	0.731	0.931	1.000

To comprehensively measure the connectedness of independent directors, this paper uses principal component analysis to construct a composite score as the firm-level measure of director networks (*Networks*). Panel B of Table 2 reports the principal component analysis for the four centrality degrees. The factor score from the first principal component with eigenvalues greater than 1 is used to measure the director networks’ connectedness.

## Empirical result

### Descriptive statistics

Table 3 provides descriptive statistics for the 31,826 firm-year observations. As shown in Panel A, the 25th and 75th quartiles of *Tunnel* range from 0.004 to 0.022, indicating that tunneling is prevalent in the sample companies. Also, the minimum value of *Tunnel* is 0.000 and the maximum value is 0.246, which suggests that the degree of appropriation by controlling shareholders varies considerably among companies. In Panel B, the sample is divided into the high-connected group and the low-connected group based on the median of *Networks*, and the differences in key variables between the two groups are compared. As shown in Panel B, in the low-connected group, the mean value of *Tunnel* was 0.023. In the high-connected group, the mean value of *Tunnel* was 0.021. The mean difference test between the two groups was significant at the 1% level. This is consistent with the previous analysis, where controlling shareholders’ tunneling behavior exhibits greater variation among companies with different degrees of director network connectedness. In the high connected group, controlling shareholders exhibit lower tunneling behavior, which implies that director networks inhibit tunneling behavior to some extent, Hypothesis is initially proved. In the correlation analysis of Panel C, the three proxy variables of Networks, *Networks_media*, *Networks_mean,* and *Networks_max* all present significantly negative correlations with *Tunnel*, consistent with the Hypothesis.

**Table 3:** Descriptive statistics.

**Panel A: Descriptive statistics of the full sample**								
Variable	N	Mean	Min	P25	P50	P75	Max	SD
*Tunnel*	31,826	0.022	0.000	0.004	0.009	0.022	0.246	0.038
*Size*	31,826	22.069	19.310	21.160	21.911	22.810	26.027	1.294
*ROA*	31,826	0.030	−0.358	0.011	0.033	0.062	0.197	0.073
*Growth*	31,826	0.174	−0.650	−0.033	0.105	0.271	2.969	0.459
*Loss*	31,826	0.117	0.000	0.000	0.000	0.000	1.000	0.322
*Lev*	31,826	0.463	0.062	0.301	0.460	0.616	1.010	0.211
*CurrentRatio*	31,826	2.061	0.224	1.020	1.463	2.279	12.913	1.982
*Dual*	31,826	0.230	0.000	0.000	0.000	0.000	1.000	0.421
*Ratio*	31,826	0.371	0.286	0.333	0.333	0.400	0.571	0.053
*BTM*	31,826	0.324	−0.005	0.208	0.304	0.421	0.787	0.162
*SOE*	31,826	0.203	0.000	0.000	0.000	0.000	1.000	0.402
*InvestorShare*	31,826	0.465	0.005	0.299	0.489	0.643	0.907	0.232
*Big4*	31,826	0.059	0.000	0.000	0.000	0.000	1.000	0.236
**Panel B: Difference analysis between groups**								
	Low-connected		High-connected			Difference		
	N	Mean	N		Mean			
*Tunnel*	15,919	0.023	15,907		0.021	0.003^***^		
*Size*	15,919	21.873	15,907		22.265	−0.393^***^		
*ROA*	15,919	0.027	15,907		0.033	−0.007^***^		
*Growth*	15,919	0.171	15,907		0.177	−0.006		
*Loss*	15,919	0.134	15,907		0.101	0.033^***^		
*Lev*	15,919	0.453	15,907		0.473	−0.020^***^		
*CurrentRatio*	15,919	2.175	15,907		1.947	0.229^***^		
*Dual*	15,919	0.247	15,907		0.213	0.033^***^		
*Ratio*	15,919	0.372	15,907		0.369	0.004^***^		
*BTM*	15,919	0.320	15,907		0.328	−0.008^***^		
*SOE*	15,919	0.183	15,907		0.222	−0.039^***^		
*InvestorShare*	15,919	0.444	15,907		0.485	−0.041^***^		
*Big4*	15,919	0.047	15,907		0.072	−0.024^***^		
**Panel C: Correlation analysis**								
	(1)	(2)	(3)	(4)	(5)	(6)	(7)	(8)
(1) *Tunnel*	1							
(2) *Networks_media*	−0.017^***^	1						
(3) *Networks_mean*	−0.019^***^	0.923^***^	1					
(4) *Networks_max*	−0.031^***^	0.805^***^	0.962^***^	1				
(5) *Size*	−0.126^***^	0.142^***^	0.152^***^	0.164^***^	1			
(6) *ROA*	−0.270^***^	0.063^***^	0.074^***^	0.076^***^	0.099^***^	1		
(7) *Growth*	−0.041^***^	0.024^***^	0.029^***^	0.027^***^	0.033^***^	0.228^***^	1	
(8) *Loss*	0.196^***^	−0.061^***^	−0.069^***^	−0.069^***^	−0.119^***^	−0.690^***^	−0.199^***^	1
(9) *Lev*	0.226^***^	0.077^***^	0.086^***^	0.083^***^	0.336^***^	−0.375^***^	0.025^***^	0.224^***^
(10) *CurrentRatio*	−0.092^***^	−0.074^***^	−0.085^***^	−0.082^***^	−0.255^***^	0.212^***^	−0.030^***^	−0.116^***^
(11) *Dual*	−0.020^***^	−0.074^***^	−0.086^***^	−0.084^***^	−0.123^***^	0.007	0.006	0.007
(12) *Ratio*	−0.016^***^	−0.092^***^	−0.091^***^	−0.048^***^	0.038^***^	−0.020^***^	−0.006	0.011^**^
(13) *BTM*	−0.139^***^	−0.006	−0.007	−0.009	0.072^***^	0.152^***^	−0.052^***^	−0.165^***^
(14) *SOE*	0.056^***^	0.116^***^	0.148^***^	0.143^***^	0.059^***^	−0.007	0.048^***^	−0.017^***^
(15) *InvestorShare*	−0.008	0.146^***^	0.170^***^	0.168^***^	0.366^***^	0.114^***^	0.054^***^	−0.088^***^
(16) *Big4*	−0.034^***^	0.074^***^	0.093^***^	0.106^***^	0.333^***^	0.059^***^	−0.015^***^	−0.042^***^
	(9)	(10)	(11)	(12)	(13)	(14)	(15)	(16)
(9) *Lev*	1							
(10) *CurrentRatio*	−0.646^***^	1						
(11) *Dual*	−0.118^***^	0.118^***^	1					
(12) *Ratio*	−0.019^***^	0.035^***^	0.121^***^	1				
(13) *BTM*	−0.536^***^	0.262^***^	−0.008	−0.042^***^	1			
(14) *SOE*	0.130^***^	−0.130^***^	−0.157^***^	−0.101^***^	0.068^***^	1		
(15) *InvestorShare*	0.176^***^	−0.171^***^	−0.210^***^	−0.084^***^	−0.020^***^	0.290^***^	1	
(16) *Big4*	0.062^***^	−0.075^***^	−0.064^***^	0.025^***^	0.053^***^	0.057^***^	0.236^***^	1

### Main analyses

This paper first examines the relationship between director networks and tunneling. Table 4 reports the regression results. In column (1), the result shows a negative and significant coefficient for *Networks_media* (−2.61, *p* < 0.01). When the regression analysis is re-run using *Networks_mean* (−3.60, p < 0.01) and *Networks_max* (−4.22, p < 0.01) as independent variables, the results are robust. These results suggest that firms with well-connected director networks have less tunneling behavior. Following Omer et al. (2020), this paper also calculates the economic significance. For one standard deviation change in *Networks*^1^, the odds of a decrease in tunneling behavior are 12.6, 12.8, and 13.1%, respectively. The impact of the director networks on tunneling behavior is also economically significant. In summary, Table 4 supports the view that the degree of independent director connectedness is associated with lower controlling shareholders’ tunneling behavior.

**Table 4:** Multivariate results for director networks and tunneling.

	(1)	(2)	(3)
*Networks_media*	−0.001^***^		
	(−3.99)		
*Networks_mean*		−0.001^***^	
		(−5.63)	
*Networks_max*			−0.001^***^
			(−6.52)
*Size*	−0.003^***^	−0.002^***^	−0.002^***^
	(−9.41)	(−9.13)	(−8.98)
*ROA*	−0.083^***^	−0.083^***^	−0.083^***^
	(−12.60)	(−12.57)	(−12.56)
*Growth*	−0.001^*^	−0.001^*^	−0.001^*^
	(−1.66)	(−1.69)	(−1.70)
*Loss*	0.003^**^	0.003^**^	0.003^**^
	(2.44)	(2.44)	(2.44)
*Lev*	0.029^***^	0.029^***^	0.029^***^
	(12.33)	(12.27)	(12.24)
*CurrentRatio*	0.001^***^	0.001^***^	0.001^***^
	(10.44)	(10.39)	(10.36)
*Dual*	0.000	−0.000	−0.000
	(0.02)	(−0.02)	(−0.05)
*Ratio*	0.004	0.004	0.005
	(1.17)	(1.09)	(1.32)
*BTM*	−0.016^***^	−0.016^***^	−0.016^***^
	(−8.69)	(−8.79)	(−8.85)
*SOE*	−0.005^***^	−0.005^***^	−0.005^***^
	(−7.64)	(−7.58)	(−7.52)
*InvestorShare*	−0.002^***^	−0.002^***^	−0.002^***^
	(−2.69)	(−2.66)	(−2.65)
*Big4*	0.000	0.000	0.000
	(0.29)	(0.36)	(0.47)
_cons	0.116^***^	0.114^***^	0.113^***^
	(18.74)	(18.54)	(18.35)
Year Fixed	Yes	Yes	Yes
Industry Fixed	Yes	Yes	Yes
N	31,826	31,826	31,826
Adj. R-Square	0.214	0.214	0.215

^***^, ^**^, and ^*^ denote significance at the 1, 5, and 10% level, respectively.

Consistent with previous research (Gao and Kling, 2008; Liu et al., 2016), controlling shareholders’ tunneling behavior increases with poor company performance (*Loss*), financial leverage (*Lev*), and current ratio (*CurrentRatio*). This paper finds negative and significant coefficients for *Size*, *ROA*, *Growth*, *BTM*, *SOE,* and *InvestorShare*, indicating that firms with a larger size, higher profitability, and better growth opportunities are less likely to experience controlling shareholders’ expropriation. In addition, state-owned enterprises and firms with higher shareholdings of institutional investors have fewer controlling shareholders’ tunneling behavior.

### Robustness tests

To further investigate the robustness of the results, this paper conducts a series of tests. Firstly, following Liu et al. (2016), the paper performs the regression analysis by using ln(1 + *Tunnel*) as the dependent variable. The results are shown in Panel A of Table 5, where the coefficients of *Networks_media*, *Networks_mean*, and *Networks_max* are still significantly negative after replacing the measurement of the dependent variable. As a result, the conclusions remain consistent after changing the dependent variable’s measurements. Second, clustering standard errors at the firm level are used to settle possible clustering effects in the main regression analysis (Liu et al., 2016). The results are shown in Table 5. The coefficient of *Networks_media*, *Networks_mean*, and *Networks_max* are significantly negative, which is consistent with the results in Table 4. In conclusion, the results remain robust after adjusting for clustering effects. Third, the financial crisis severely undermines firms’ performance, making it difficult for controlling shareholders to appropriate firms’ interests. Sometimes, the controlling shareholders may be required to provide support to the company. To avoid the impact of the financial crisis, this study restricts the sample to the period before the financial crisis, that is, before 2006. In panel C of Table 5, the results are consistent with the full sample, which means that the sample contains financial crisis years that do not affect the main results. Finally, as How et al. (2008) revealed, compared to larger companies, smaller companies have more limitations in acquiring resources and suffer from worse corporate governance, which makes them easier to expropriate by controlling shareholders. If the results are influenced by firm size, then it is expected that more significant results on director networks and tunneling behavior will be observed in the small firm. To further test whether the results are driven by firm size, this paper divides the sample into two groups based on firm size quartiles. If the firm size is in the first quartile, it is in the small firm group, otherwise it is in the large firm group. As shown in Panel D of Table 5, the coefficients of *Networks_media*, *Networks_mean* and *Networks_max* are significantly negative in both sample groups. This alleviates concerns about the effect of firm size.

**Table 5:** Robustness test.

**Panel A: Changing the measurement of the dependent variable**						
	(1)		(2)		(3)	
*Networks_media*	−0.001^**^					
	(−2.49)					
*Networks_mean*			−0.001^***^			
			(−3.45)			
*Networks_max*					−0.001^***^	
					(−4.06)	
Controls	0.107^***^		0.106^***^		0.105^***^	
	(11.44)		(11.29)		(11.16)	
Year Fixed	Yes		Yes		Yes	
Industry Fixed	Yes		Yes		Yes	
Firm Fixed	Yes		Yes		Yes	
N	31,826		31,826		31,826	
Adj. R-Square	0.217		0.218		0.218	
**Panel B: Cluster analysis at the firm level**						
	(1)		(2)		(3)	
*Networks_media*	−0.001^***^					
	(−2.61)					
*Networks_mean*			−0.001^***^			
			(−3.58)			
*Networks_max*					−0.001^***^	
					(−4.19)	
Controls	Yes		Yes		Yes	
Year Fixed	Yes		Yes		Yes	
Industry Fixed	Yes		Yes		Yes	
N	31,826		31,826		31,826	
Adj. R-Square	0.214		0.214		0.215	
**Panel C: Avoiding the impact of the financial crisis**						
	(1)		(2)		(3)	
*Networks_media*	−0.002^***^					
	(−3.79)					
*Networks_mean*			−0.002^***^			
			(−3.18)			
*Networks_max*					−0.002^***^	
					(−2.71)	
Controls	Yes		Yes		Yes	
Year Fixed	Yes		Yes		Yes	
Industry Fixed	Yes		Yes		Yes	
N	2,820		2,820		2,820	
Adj. R-Square	0.403		0.403		0.402	
**Panel D: The impact of firm size**						
	(1) Small	(2) Large	(3) Small	(4) Large	(5) Small	(6) Large
*Networks_media*	−0.001^***^	−0.000^*^				
	(−3.14)	(−1.71)				
*Networks_mean*			−0.002^***^	−0.000^***^		
			(−3.71)	(−2.94)		
*Networks_max*					−0.002^***^	−0.001^***^
					(−3.79)	(−4.08)
Controls	Yes	Yes	Yes	Yes	Yes	Yes
Year Fixed	Yes	Yes	Yes	Yes	Yes	Yes
Industry Fixed	Yes	Yes	Yes	Yes	Yes	Yes
N	7,957	23,869	7,957	23,869	7,957	23,869
Adj. R-Square	0.320	0.141	0.320	0.141	0.321	0.142

^***^, ^**^, and ^*^ denote significance at the 1, 5, and 10% level, respectively.

### Endogeneity tests

In the above analysis, this paper has demonstrated that well-connected independent directors play an important role in mitigating controlling shareholders’ tunneling behavior. However, some omitted variables may lead to a spurious negative correlation between director networks and tunneling behavior. To mitigate this concern, this paper first controls for firm fixed effects in the baseline regressions to control for the impact of time-invariant firm characteristics on the results. The results are shown in Panel A of Table 6. The coefficients of *Networks_media*, *Networks_mean*, and *Networks_max* are significantly negative. The results support the findings that director networks inhibit controlling shareholders’ tunneling behavior.

**Table 6:** Endogeneity tests.

**Panel A: Control for firm-level fixed effects**			
	(1)	(2)	(3)
*Networks_media*	−0.000^*^		
	(−1.72)		
*Networks_mean*		−0.001^***^	
		(−2.90)	
*Networks_max*			−0.001^***^
			(−3.73)
Controls	Yes	Yes	Yes
Year Fixed	Yes	Yes	Yes
Industry Fixed	Yes	Yes	Yes
N	31,826	31,826	31,826
Adj. R-Square	0.039	0.039	0.039
**Panel B: Impact of official independent directors’ departure**			
	(1)	(2)	(3)
*Post*	−0.045^***^	−0.045^***^	−0.045^***^
	(−12.75)	(−12.74)	(−12.85)
*High_med*	−0.002^**^		
	(−2.00)		
High_med^*^Post	0.003^***^		
	(3.40)		
*High_ mean*		−0.002^***^	
		(−3.47)	
High_mean^*^Post		0.002^***^	
		(3.14)	
*High_max*			−0.003^***^
			(−5.10)
High_max^*^Post			0.003^***^
			(3.59)
Controls	Yes	Yes	Yes
Year Fixed	Yes	Yes	Yes
Industry Fixed	Yes	Yes	Yes
N	31,826	30,465	31,826
Adj. R-Square	0.214	0.216	0.214
**Panel C: Changes in director networks**			
	(1)	(2)	(3)
	Δ*Networks_media*	Δ*Networks_mean*	Δ*Networks_max*
*Lag_Occupy*	−0.207	−0.238	−0.220
	(−1.14)	(−1.34)	(−1.13)
*Lag_Controls*	Yes	Yes	Yes
Year Fixed	Yes	Yes	Yes
Industry Fixed	Yes	Yes	Yes
N	31,826	30,465	31,826
Adj. R-Square	0.214	0.216	0.214

^***^, ^**^, and ^*^ denote significance at the 1, 5, and 10% level, respectively.

Secondly, controlling shareholders retain considerable control over the company, they may interfere with the appointment of independent directors and choose the “obedient” independent directors to join the company to successfully conduct tunneling. In addition, as mentioned above, well-connected independent directors usually possess a higher reputation, which makes them more likely to accept offers from companies in good standing. To alleviate the potential endogeneity issues affecting the results, this paper addresses the impact of director networks on tunneling behavior in an exogenous shock context. On 19 October 2013, the Organization Department of the CPC issued the “Opinions on Further Regulating the Issue of Party and Government Leading Cadres’ Part-time Positions in Enterprises” (hereafter No. 18), which prohibited officials from serving as independent directors in listed companies. No. 18 triggered many independent directors with officer status to resign in subsequent years. If well-connected independent directors play an important role in restraining controlling shareholders’ tunneling behavior, then the paper expects that after the issuance of No. 18, tunneling behavior in companies with well-connected director networks will increase. *Post* is set to 1 for the year immediately after the No. 18 and 0 otherwise. In addition, following Fang et al. (2021), *High_med* (calculated according to *Networks_media*) is equal to 1 for director networks above the industry median and 0 otherwise (the similar setting for *High_mean* and *High_max*). As shown in Panel B of Table 6, the coefficient on High_med^*^Post (3.40, *p* < 0.01) is significantly positive. Using *High_mean* and *High_max*, the conclusion stands. The results indicate that controlling shareholders’ tunneling behavior increases when companies lose many broadly connected independent directors.

Third, as analyzed above, independent directors with rich social networks may avoid companies with heavy tunneling behavior due to reputational concerns. To address this issue, the paper uses the changes in *Networks* from *t*-1 to *t* (Δ*Networks_media*, Δ*Networks_mean*, and Δ*Networks_max*) as the dependent variable, *Occupy* with one lag as the independent variable (*Lag_Occupy*), and controls for the lagged values of all the above control variables. If this concern holds, then it should be possible to observe a significantly negative coefficient on *Lag_Occupy* in this regression. In Panel C of Table 6, the coefficient of *Lag_Occupy* is not significant in any of the three groups. Generally, the paper does not find any evidence that director networks are endogenously matched to controlling shareholders’ tunneling behavior.

## The mediating effect of internal control

In the above analysis, the paper finds that director networks significantly inhibit controlling shareholders’ tunneling behavior. One possible explanation is that well-connected independent directors, with the information gained from the director networks and extensive experience accumulated from serving in several companies, help to improve the quality of internal controls, which in turn inhibits controlling shareholders’ opportunistic behavior. To test this conjecture, the research introduces the mediating variable of internal control (*IC*). By constructing the mediating effect model, the paper analyses whether director networks inhibit controlling shareholders’ tunneling behavior by improving the quality of internal control. Following Chan et al. (2021), the paper uses the Internal Control Disclosure Index (in natural logarithms) from the DIB Internal Control and Risk Management Database to measure internal control quality (*IC*). A higher value of *IC* indicates that the company has higher internal control quality. In the baseline regressions, the results have demonstrated the general effect of director networks on controlling shareholders’ tunneling behavior. Next, the paper further tests the mediating effect of internal control by constructing the following two equations:


(2) $$\begin{array}{c} IC_{i,t}=\alpha_{0}+\alpha_{1}Networks_{i,t}+Controls_{i,t}+\\ \sum Year+\sum Industry+\varepsilon_{i,t} \end{array}$$

(3) $$\begin{array}{c} Tunnel_{i,t}=\lambda_{0}+\lambda_{1}Networks_{i,t}+\lambda_{2}IC_{i,t}+\\ Controls_{i,t}+\sum Year+\sum Industry+\varepsilon_{i,t} \end{array}$$

Columns (1)–(3) in Table 7 show the relationship between director networks and internal control. The coefficients of *Networks_media*, *Networks_mean,* and *Networks_max* are all significantly positive at the 1% level, implying that enriched social networks of independent directors contribute to companies’ internal control quality. This result is consistent with Sun et al. (2012) that independent directors play an important role in improving internal control quality. Columns (4)–(6) of Table 7 show the results after adding the mediating variable *IC*. The coefficients of *Networks_media*, *Networks_mean,* and *Networks_max* remain significant after adding the mediating variable *IC* to the baseline analysis, which indicates that the mediating effect of internal control holds. Ge et al. (2021) provided evidence that high-quality internal controls are effective in reducing controlling shareholders’ resource extraction and protecting the interests of minority shareholders. Based on this perspective, this paper provides further evidence that well-connected independent directors play a positive role in improving internal control quality in companies, which in turn effectively restrains controlling shareholders’ tunneling behavior.

**Table 7:** Mediating effect.

	(1)	(2)	(3)	(4)	(5)	(6)
	*IC*	*IC*	*IC*	*Occupy*	*Occupy*	*Occupy*
*Networks_media*	0.004^***^			−0.000^**^		
	(6.22)			(−2.28)		
*Networks_mean*		0.005^***^			−0.001^***^	
		(7.67)			(−3.55)	
*Networks_max*			0.005^***^			−0.001^***^
			(7.87)			(−4.37)
*IC*				−0.024^***^	−0.024^***^	−0.024^***^
				(−11.69)	(−11.66)	(−11.64)
Controls	Yes	Yes	Yes	Yes	Yes	Yes
Year Fixed	Yes	Yes	Yes	Yes	Yes	Yes
Industry Fixed	Yes	Yes	Yes	Yes	Yes	Yes
N	30,402	30,402	30,402	30,402	30,402	30,402
Adj. R-Square	0.297	0.298	0.298	0.226	0.226	0.227

^***^, ^**^, and ^*^ denote significance at the 1, 5, and 10% level, respectively.

## Cross-sectional analyses

While the above analysis provides evidence that director networks are significantly and negatively related to controlling shareholders’ tunneling behavior, this paper conjectures that the effect of director networks on tunneling behavior will vary for firms with different ownership types as well as governance environments. Especially in firms with weak governance, the restraining effect of director networks on tunneling behavior is more remarkable. Therefore, the following two sets of cross-sectional analyses are carried out: (1) the degree of the audit firm’s monitoring; (2) the degree of firm transparency.

### Quality of audit monitoring

Previous studies show that audit plays an important role in corporate governance (Ashbaugh and Warfield, 2003). Gao and Kling (2008) and Jiang et al. (2010) also find that auditors can effectively monitor tunneling behavior and companies with a high degree of tunneling are more likely to receive a qualified opinion from the auditor. Therefore, this paper argues that the restraining effect of director networks on tunneling behavior is not significant in companies with higher quality audit monitoring. Previous studies have shown that Big 4 audit firms provide higher quality audits compared to non-Big 4 audit firms (Khurana and Raman, 2004; De Franco et al., 2011). Big 4 auditors play a more effective external monitoring role in corporate governance (Fan and Wong, 2005; Gul et al., 2010). Consequently, following Gul et al. (2010), the sample is divided into two groups based on whether the company is audited by international Big 4 audit firms. That is, *Big4* equals 1 if the company is audited by Big 4 audit firms and 0 otherwise. It is expected that the effect of director networks on tunneling behavior is more significant in companies audited by non-Big 4. The results are shown in columns (1) and (2) of Table 8. In the non-Big4 audit sample, the coefficient on *Networks* (−3.60, *p* < 0.01) is significantly negative. While in the Big4 group, the coefficient of *Networks* is not significant. As expected, director networks have more significant inhibitory effects on tunneling behavior in companies with poorer audit governance.

**Table 8:** Cross-sectional analyses.

	(1)	(2)	(3)	(4)
	*Big4* = 1	*Big4* = 0	*Opacity* = 1	*Opacity* = 0
*Networks*	0.000	−0.001^***^	0.000	−0.001^***^
	(0.42)	(−3.60)	(1.10)	(−4.21)
Controls	Yes	Yes	Yes	Yes
Year Fixed	Yes	Yes	Yes	Yes
Industry Fixed	Yes	Yes	Yes	Yes
	Prob > chi2 = 0.052		Prob > chi2 = 0.000	
N	1892	29,934	17,612	14,214
Adj. R-Square	0.202	0.219	0.159	0.224

^***^, ^**^, and ^*^ denote significance at the 1, 5, and 10% level, respectively.

### Corporate transparency

Bhat et al. (2006) provide evidence that companies with a high level of transparency exhibit stronger corporate governance, which in turn helps analysts issue more accurate earnings forecasts. Improving corporate transparency also helps promote the efficient allocating of resources (Francis et al., 2009), mitigate the impact of investor sentiment on share prices (Firth et al., 2015), as well as reduce IPO costs (Ang and Brau, 2002). For this reason, this paper argues that companies with high transparency have low information asymmetry with external stakeholders, where the effect of director networks on tunneling behavior is not significant. In contrast, controlling shareholders’ tunneling behavior is more likely to occur in companies with low transparency, and the governance effect of director networks is more pronounced. The Shanghai Stock Exchange and Shenzhen Stock Exchange annually evaluate the information disclosure work of listed companies, which is divided into four grades from high to low: A, B, C, and D. Companies with A or B results are classified in the high disclosure group, and *Opacity* equals 1.^2^ The remaining sample is in the low information disclosure group, *Opacity* equals to 0. The regression results are shown in columns (3) and (4) of Table 8. Consistent with the prediction, the restraining effect of director networks on controlling shareholders’ tunneling behavior is only significant in the group with lower transparency.

## Conclusion

This paper examines how well-connected independent directors influence controlling shareholders’ tunneling behavior. Studies based on social network theory argue that social networks built by independent directors through serving on multiple boards help them to access and pass on information, which is essential for improving independent directors’ corporate governance capabilities. In accordance with this perspective, the paper proposes the hypothesis that director networks have a negative relationship with controlling shareholders’ tunneling behavior.

Using a sample of Chinese listed companies from 2002 to 2020, this paper provides evidence of how director networks influence controlling shareholders’ tunneling behavior. The results show that well-connected director networks contribute to independent directors’ governance capacity, which helps to inhibit controlling stakeholders’ expropriation of minority shareholders. This paper reports the same results through robustness tests, such as replacing the measurement of the dependent variable, cluster analysis at the firm level, and narrowing the sample, as well as endogeneity tests, including controlling for firm-level fixed effects and using officer director departures as exogenous events. Overall, the results indicate that external networking of independent directors can help curb controlling shareholders’ tunneling behavior and protect minority shareholders’ interests. The Hypothesis is verified. Further, the analysis shows that internal control plays a mediating role. Well-connected independent directors help to improve the firms’ internal control quality, which in turn reduces the risk of controlling shareholders engaging in tunneling behavior. In addition, cross-sectional analyses show that the relationship between director networks and controlling shareholder tunneling behavior is more pronounced in companies with poor external audit oversight and less transparency. Overall, the results suggest that extensive director networks strengthen independent directors’ capacity in corporate governance.

This paper has some implications. The results have shown that companies benefit from independent directors’ networking. Well-connected independent directors not only protect the company’s sustainability and minority shareholders’ interests but also compensate for the inadequacy of other governance mechanisms. Therefore, when appointing independent directors, companies can broadly consider the network location of the independent director and the resources they can bring to the company.

There are limitations to this study. Although this paper adopts some research design to alleviate the endogeneity matching issue between director networks and tunneling behavior, it is difficult to completely eliminate the influence of omitted variables. Secondly, the sample in this paper includes only listed companies. Unlisted companies account for a large proportion of the Chinese capital market. Moreover, compared to listed companies, unlisted companies face less strict regulation, and controlling shareholders are more likely to engage in tunneling. However, owing to limitations on data availability, this paper is restricted to listed companies. Consequently, the results in this paper should be interpreted with caution. If the data allow, future research could discuss whether the negative relationship between director networks and controlling shareholder tunneling behavior also holds in unlisted companies. Cross-country studies are also recommended. Through multi-country studies, it is further investigated whether director networks differ in governance effectiveness in different countries and different institutional contexts.

## Data availability statement

Publicly available datasets were analyzed in this study. This data can be found at: https://www.gtarsc.com/.

## Author contributions

HC and JW participated in the study design, developed the theoretical framework, and wrote the first draft of the manuscript. MX was responsible for data collection and analysis. HC and JW reviewed the manuscript. All authors contributed to the article and approved the submitted version, led by HC.

## Funding

The authors gratefully thank the financial support provided by the Postgraduate Research & Practice Innovation Program of Jiangsu Province (KYCX21_0392).

## Conflict of interest

The authors declare that the research was conducted in the absence of any commercial or financial relationships that could be construed as a potential conflict of interest.

## Publisher’s note

All claims expressed in this article are solely those of the authors and do not necessarily represent those of their affiliated organizations, or those of the publisher, the editors and the reviewers. Any product that may be evaluated in this article, or claim that may be made by its manufacturer, is not guaranteed or endorsed by the publisher.

## Figures and Tables

**Table:** 

Variable	Definition
*Tunnel*	Other receivables divided by total assets
*Networks*	Comprehensive scores for *Degree*, *Closeness*, *Betweenness* and *Eigenvector* calculated by principal component analysis. In particular, *Networks_media*, *Networks_mean* and *Networks_max* are the company-level network connectedness calculated based on the median, mean and maximum values of the independent directors’ network indicators
*Size*	The natural logarithm of total assets
*ROA*	Net income divided by total assets
*Growth*	Growth in sales for the period compared to sales in the last period
*Loss*	1 if net profit is less than zero, otherwise 0
*Lev*	Total liabilities divided by total assets
*CurrentRatio*	Current assets divided by current liabilities
*Dual*	1 if the chairman and CEO of the company are the same person, otherwise 0
*Ratio*	Percentage of independent directors on the board
*BTM*	Book-to-market ratio
*SOE*	1 if the firm’s ultimate controller is a government-owned entity, otherwise 0
*IC*	The natural logarithm of the Internal Control Index score
*InvestorShare*	Shares held by institutional investors
*Big4*	1 if the firm is audited by a Big 4 international audit firm, otherwise 0
*Opacity*	1 if annual corporate transparency rating is A or B, otherwise 0

## Footnotes

^1^In the untabulated analysis, the standard deviation δ of *Networks_medi*a, *Networks_mean* and *Networks_max* were 1.263, 1.284 and 1.309, respectively. According to the equation, $100*\left(e^{\beta_{k}*\delta }-1\right)$ , the economic magnitude of the three coefficients are −0.126, −0.128 and − 0.131, respectively.

^2^In the untabulated analysis, this paper defines the group with result A as the high disclosure group, while B,C and D are classified as the low disclosure group, the results are consistent.
